# Short echo-time Magnetic Resonance Spectroscopy in ALS, simultaneous quantification of glutamate and GABA at 3 T

**DOI:** 10.1038/s41598-019-53009-4

**Published:** 2019-11-26

**Authors:** J. U. Blicher, S. F. Eskildsen, T. G. Stærmose, A. T. Møller, K. Figlewski, J. Near

**Affiliations:** 1 Center of Functionally Integrative Neuroscience (CFIN), Aarhus University, Aarhus, Denmark; 2 Department of Neurology, Aarhus University Hospital, Aarhus, Denmark; 3 Department of Psychiatry, McGill University, Montreal, Canada

**Keywords:** Prognostic markers, Amyotrophic lateral sclerosis

## Abstract

Cortical hyperexcitability has been found in early Amyotrophic Lateral Sclerosis (ALS) and is hypothesized to be a key factor in pathogenesis. The current pilot study aimed to investigate cortical inhibitory/excitatory balance in ALS using short-echo Magnetic Resonance Spectroscopy (MRS). Patients suffering from ALS were scanned on a 3 T Trio Siemens MR scanner using Spin Echo Full Intensity Acquired Localized (SPECIAL) Magnetic Resonance Spectroscopy in primary motor cortex and the occipital lobe. Data was compared to a group of healthy subjects. Nine patients completed the scan. MRS data was of an excellent quality allowing for quantification of a range of metabolites of interest in ALS. In motor cortex, patients had Glutamate/GABA and GABA/Cr- ratios comparable to healthy subjects. However, Glutamate/Cr (p = 0.002) and the neuronal marker N-acetyl-aspartate (NAA/Cr) (p = 0.034) were low, possibly due to grey-matter atrophy, whereas Glutathione/Cr (p = 0.04) was elevated. In patients, NAA levels correlated significantly with both hand strength (p = 0.027) and disease severity (p = 0.016). In summary SPECIAL MRS at 3 T allows of reliable quantification of a range of metabolites of interest in ALS, including both excitatory and inhibitory neurotransmitters. The method is a promising new technique as a biomarker for future studies on ALS pathophysiology and monitoring of disease progression.

## Introduction

Amyotrophic Lateral Sclerosis (ALS) is a devastating neurodegenerative disease leading to severe disability, followed by death within a few years of diagnosis. Development of a cure for ALS is hampered by the lack of understanding of the underlying pathophysiology, and the lack of good biomarkers to measure the effect of candidate drugs on cortical disease progression. Cortical hyperexcitability, as measured by Transcranial Magnetic Stimulation (TMS), is a feature of early ALS^1^ and is hypothesized to be a key factor in disease spread from the brain to the spinal cord, a model commonly referred to as the ‘dying forward’ model. In later stages of ALS, TMS measures of cortical excitability are more difficult to interpret as the measured motor evoked potentials are attenuated due to muscle atrophy. Thus, TMS measures of cortical excitability are useable in early detection of cortical involvement in ALS, however the technique is less useful as a biomarker to track disease progression, or as an outcome measure in clinical trials investigating candidate therapeutics against ALS. Magnetic Resonance Spectroscopy (MRS) is a technique capable of quantifying metabolite concentrations in the brain, and has shown some potential in detecting neurochemical abnormalities associated with ALS. There is a wealth of MRS studies in ALS^2,3^ (see^4^ for an overview), and several have shown low N-acetylaspartate (NAA), interpreted as neuronal loss or impairment^5,6^. Although the exact role of NAA in the brain is still not clarified it is often interpreted as a marker of neuronal integrity, however a decline in NAA does not necessarily indicate neuronal death^7^ and NAA has been shown to increase after start of Riluzole therapy^8^. More recently, developments in scanner hardware and MRS data acquisition, processing and analysis methods have enabled quantification of a greater number of brain metabolites, including relevant neurotransmitters such as glutamate and γ-aminobutyric acid (GABA). Two MRS studies at 3 T reported low cortical GABA levels in ALS using a MEGA-PRESS edited sequence^9,10^ whereas a recent 7 T MRS study found low glutamate and no changes in GABA^11^. Here we report the results of a pilot MRS study in a small group of ALS patients using a novel short echo-time MRS technique allowing for detection of a relatively large number of metabolites simultaneously^12^. The aim of the study was to investigate alterations in neurochemical concentrations in patients with ALS, compared with healthy controls to clarify whether the sequence would be useful for larger longitudinal studies in ALS. Our pre-study hypotheses were:

1. That the Glutamate/GABA ratio would be high in ALS patients compared to healthy subjects, as suggested in prior studies^9,10^
2. NAA levels would be reduced in ALS patients relative to healthy subjects^5,6^.
  The secondary aims of this study were to investigate changes in other metabolites, including *myo*-inositol (mIns), choline (Cho) and Glutathione (GSH), and to test the association between MRS and clinical outcome measures.

## Results

### Magnetic resonance spectroscopy data quality

**Figure 1:**
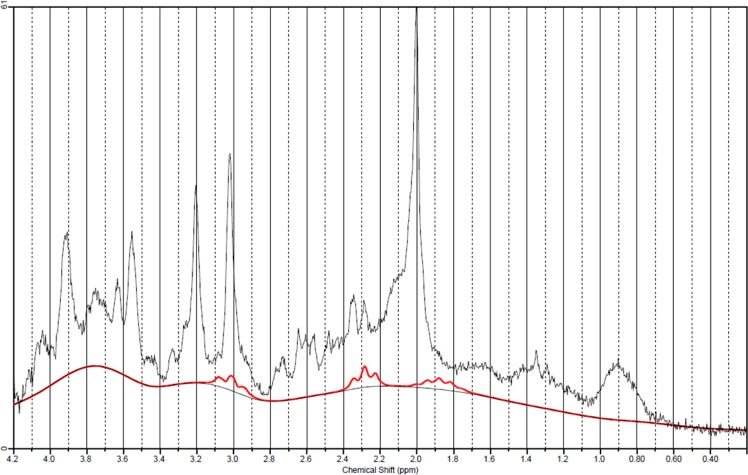
A typical short-echo MRS spectrum from the motor cortex of an ALS patient. The spectrum is in black and the fitted GABA peaks are in red (1.9, 2.3 and 3.0 ppm).

The MRS data was consistently of excellent quality (see Fig. 1 for a typical patient example). In the motor voxel the mean SNR (as reported from LCModel) was 45 (range 33–56) in patients and 46 (range 39–56) in healthy subjects. The Full width at half maximum (FWHM)of the water peak did not differ significantly (p = 0.10) between patients (0.0262) and healthy subjects (0.0296). The estimated uncertainty on metabolite estimates (CRLB) was generally low for Cr + PCr (patients mean = 2%, healthy subjects mean = 2%), GABA (patients mean = 12.4%, healthy mean = 12.2%), Glutamine (patients mean = 13.6%, healthy subjects mean = 15.2%), Glutamate (patients mean = 4.8%, healthy subjects mean = 4.4%), GSH (patients mean = 7.1%, healthy subjects mean = 8.7%), mIns (patients mean = 4.1%, healthy subjects mean = 4.8%) and tNAA (patients mean = 1.4%, healthy subjects mean = 1.4%). In all metabolites of interest CRLB was below the 20% cut-off for each subject.

In the occipital voxel the average SNR was 51 (range 38–67). CRLB uncertainties on metabolite estimates were generally low for Cr + PCr (mean = 2.9%), GABA (mean = 13.4%), Glutamine (mean = 12.7%), Glutamate (mean = 4.7%), GSH (mean = 7.7%), mIns (mean = 4.3%) and tNAA (mean = 1.4%). In one patient the CRLB was 26% for GABA.

### Differences between subjects with ALS and healthy subjects

We found no significant difference in Glutamate/GABA ratios between ALS patients and healthy subjects, although patients did show a tendency towards lower Glutamate/GABA ratios (p = 0.07). The tendency was explained by a low Glutamate/Cr ratio in patients (mean 0.81) compared to healthy subjects (mean 0.98, p = 0.002), whereas GABA/Cr and Glutamine/Cr levels were comparable between the groups (see Table 1). Moreover, patients had significantly lower tNAA/Cr (p = 0.034) and higher GSH/Cr (p = 0.04) compared to healthy subjects.

**Table 1:** Motor cortex metabolite ratios.

	Healthy subjects (n = 10), mean (SD)	ALS Subjects (n = 9), mean (SD)	p-value
tNAA/Cr	1.62 (0.12)	1.46 (0.18)	0.034*
Glutamate/Cr	0.983 (0.10)	0.809 (0.11)	0.002*
Glutamine/Cr	0.246 (0.05)	0.242 (0.04)	0.860
GABA/Cr	0.312 (0.06)	0.279 (0.02)	0.113
Glutathione/Cr	0.166 (0.02)	0.187 (0.02)	0.040*
*Myo*-inositol/Cr	0.723 (0.12)	0.832 (0.16)	0.100
tCho/Cr	0.203 (0.04)	0.238 (0.03)	0.042*
Cr+PCr/water	6.26 (0.52)	5.79 (0.70)	0.11

*Significant using a Students two-tailed t-test p < 0.05.

A comparison of GM content within the motor voxel showed that patients with ALS had a reduced amount of GM (26%) compared to healthy subjects (31%)(p = 0.047). In the patient group a significant positive correlation was found between Glutamate/Cr and GM-content (p = 0.028, R^2^ = 0.52) as well as Glutamate/Cr and tNAA/Cr (p = 0.003, R^2^ = 0.74), whereas tNAA/Cr and GM-content was borderline significant (p = 0.05, R^2^ = 0.44). In an ANOVA analysis with GM content as an added explanatory variable, tNAA/Cr (p = 0.22) and Glutamate/Cr (p = 0.24) did not differ significantly between patients with ALS and healthy subjects, whereas GSH/Cr still differed significantly (p < 0.01).

### Association between MRS data and clinical outcome measures

Motor cortex tNAA/Cr correlated significantly with both ALSFRSr (p = 0.016, R^2^ = 0.59) and hand muscle strength (p = 0.027, R^2^ = 0.53), with low tNAA/Cr being associated with lower ALSFRSr and lower average MRC-score. Adding GM content to the regression model for ALSFRSr showed an independent significant effect of tNAA/Cr (p = 0.004) and a tendency for GM content (p = 0.055), and that the two variables together significantly predicted ALSFRSr (p = 0.01, R^2^ = 0.79). For hand muscle strength, adding GM content to the model showed a significant independent effect of tNAA/Cr (p = 0.021), and no significant effect of GM content (p = 0.225), the two variables together significantly predicted average MRC score (p = 0.047, R^2^ = 0.64). We found no significant correlation between Glutamate/Cr and ALSFRSr or average MRC-score (both > 0.05).

Occipital NAA/Cr did not correlate with ALSFRSr or average MRC-score.

## Discussion

The current results confirm that short-echo MRS allows for quantification of several metabolites of interest in ALS, including simultaneous quantification of GABA and Glutamate.

We failed to show any significant changes in GABA in ALS subjects. This is in contrast to prior studies using an edited (MEGA-PRESS) sequence to quantify GABA^9,10^, but in line with a recent 7 T study using a short-echo modelling approach compared to the current study^11^. One possible explanation could be a lack of power to detect subtle GABA difference as both the current as well as the prior 7 T study showed a tendency for low GABA levels and were small compared to prior MRS studies. Another possible explanation is the difference in methods used. In the MEGA-PRESS sequence previously used by Foerster *et al*.^9,10^ the J-coupling between neighbouring proton groups in the GABA molecules is exploited to measure GABA through a process called spectral editing. However, MEGA-PRESS normally results in co-editing of macromolecule signals, causing the measured GABA concentration to be overestimated by as much as 50%^13,14^. As a result, the MEGA-PRESS results could overestimate the decline in GABA if the amount of macromolecule contribution is low. In the current study macromolecules are modelled separately and thus the measured GABA signal maybe be less likely to be affected by changes in macromolecules.

With the short-echo approach used in the current study we were also able to separate Glutamate from Glutamine, which is usually reported as one combined signal, termed Glx, in 3 T MRS. Prior studies have reported high Glx levels^10,15^, but unchanged^16^ or low levels have also been reported^17^. Our findings are in agreement with recent results from two 7 T studies reporting low Glutamate levels in ALS^11,18^, also we confirm the prior finding of a correlation between tNAA and Glutamate^11^, indicating that the low Glutamate level likely is due to neuronal loss as previously suggested^11^. This explanation is supported by the correlation between GM content in the voxel and Glutamate levels observed in the current study. Intuitively, the observed low Glutamate levels in ALS argues against a cortical hyperactivity, however as the MRS measure of Glutamate measures both neurotransmitter and non-neurotransmitter pools of glutamate, it is not a direct measure of the ongoing signalling^19^. Moreover, to clarify whether Glutamate could be high in the early stages of ALS it would be important to investigate patients at a very early stage. In the recent study by Cheong *et al*. Glutamate levels were significantly lower in definite and probable ALS as compared to possible ALS^18^, suggesting the Glutamate levels are higher in early stages of ALS.

The ability to obtain simultaneous measures of Glutamate and GABA at 3 T could be very useful for evaluating the effect of treatments aimed at the suspected excitatory/inhibitory imbalance in ALS. As an example Di Lazzaro *et al*. recently published data suggesting that the clinical effect of repetitive TMS in ALS is associated strongly with the modulation of GABA^20^.

One prior study found low GSH levels in ALS^21^ using J-editing, but a later study failed to replicate the finding^18^. Surprisingly, we found a high GSH/Cr ratio in ALS patients. The difference between the current study and the initial finding of low GSH by Weiduschat *et al*. could potentially be explained by the different MRS techniques used, another possible explanation is that Creatine tended to be low in the patient group, giving rise to a higher GSH/Cr ratio.

### Correlation to clinical measures

For both hand muscle strength and ALSFRSr we found a correlation to tNAA in the hand area after correcting for GM in the voxel, whereas no correlation was found to Glutamate. tNAA seems to be more closely related to cortical damage than does grey matter atrophy, and thus the tNAA drop is either an indication of neuronal dysfunction prior to neuronal death or more sensitive to neuronal death than grey matter atrophy. The association between tNAA and ALSFRS^2,3^ and muscle strength^2^ has been reported previously in larger studies, the fact that we are able to reproduce the finding in this small cohort of patients supports the usefulness of short-echo MRS as a tool for studying altered neurochemistry in ALS. Moreover, the correlation between NAA/Cr and disease severity was significant after correction for GM atrophy indicating that MRS provides other information regarding cortical function than can be provided by measuring GM atrophy.

### Limitations

The main limitation of this study is the low number of patients as compared to earlier and larger studies. The sample size reflects the aim for the study, i.e. to clarify the usability of short-echo MRS in ALS. It is likely that the study has been unable to detect changes in several metabolites due to lack of power. However, the positive findings of low tNAA and Glutamate and the correlation with clinical measures are likely real as they are in line with other recent findings, the results further strengthen our confidence in future use of the short-echo MRS approach in ALS.

From the outset of this study, we carefully considered whether to use Creatine referencing or absolute quantification based on the water reference. The main problem with absolute quantification is that it relies on assumed values for the concentration of water in each tissue type and the relaxation time constants (T1 and T2) of both water and metabolite in each tissue type. Thus, absolute quantification may be biased by incorrect assumptions, especially in studies of pathology where these assumptions may not be valid. Creatine referencing on the other hand, is a simpler approach that relies on far fewer assumptions. The main assumption with the Creatine referencing method is that creatine concentrations are not significantly different between groups, and this assumption is supported by our data. Consequently, we have decided to report creatine referenced metabolite ratios, rather than estimated absolute metabolite concentration in the current manuscript.

As ALS leads to cortical atrophy, any changes in metabolite signals measured by MRS are potentially biased by disease-related changes in grey matter content in the voxel. Adjusting the metabolite concentration estimates for changes in grey matter content is not straightforward. Not only does it require knowledge of the volume fractions of gray matter within the voxel, but it also requires some knowledge of the GM:WM concentration ratio for each metabolite. Unfortunately, the latter information is not known. Any correction would require us to use an assumed GM:WM concentration ratio for each metabolite, which we unfortunately cannot do with confidence. For this reason, we have opted to report concentrations that are uncorrected for the GM content within the voxel, and use post-hoc statistical tests to ensure that the individual differences in the observed metabolite concentrations are not driven by the volume fraction of GM within the voxel.

On the other hand, the chosen method of creatine referencing may inherently provide some degree of tissue correction. That is because creatine concentrations are known to be slightly higher in GM than in WM, and nearly non-existent in CSF. Since this same (or similar) spatial distribution is observed in many of the metabolites of interest, creatine referenced metabolite ratios may be inherently less sensitive to changes in grey matter content within the voxel.

Another drawback of the study is the lack of prospectively recruited healthy subjects(see Methods). This was due to an unforeseen scanner failure, and as the gradients of the scanner had to be replaced we found that the best approach was to compare to a recently scanned group of healthy subjects recruited for another study. As an identical MRS sequence and voxel placement and size were used we are confident that any biases from this approach are minor.

## Conclusion

Short-echo MRS at 3 T is able to differentiate several metabolites of interest in ALS. With the ability to quantify GABA, Glutamate, Glutamine, Glutathione and NAA the technique is a potential useful biomarker in both diagnosis, monitoring of disease progression and as a surrogate marker in future longitudinal studies in ALS.

## Methods

The study was approved by the regional ethics committee in Region Midt (Study ID 1-1072-326-15). The study was performed in accordance with relevant guidelines/regulations (as the declaration of Helsinki). All participants gave written informed consent.

### Study participants and clinical ratings

Eleven ALS patients (9 male, mean age 65 (range 48–75)) were recruited from the Department of Neurology at Aarhus University Hospital, Denmark and scanned from February 2016 to June 2017. Inclusion criteria: Diagnosis of ALS as per the El Escorial Criteria (see table 2), age 18-90, no other severe neurological or psychiatric disease, no history of substance abuse. Patients were recruited regardless of time since diagnosis and severe of the disease. Patients were diagnosed with ALS as per the El Escorial Criteria (see Table 2). Eight patients were taking Riluzole in the recommended dose of 50 mg twice daily, one patient took 50 mg once daily (due to fatigue), and two patients did not take Riluzole. Four patients were taking SSRIs (for depression or pathological crying). Disease severity was measured using the ALSFRS-r^22^, a 12 item scale with each item rated from 0–4 (higher scores indicating better function). Muscle force was clinically rated using the 0–5 Medical Research Council (MRC) scale. An average hand muscle score was calculated from finger extension, finger flexion, thumb abduction (abductor pollicis brevis muscle), first dorsal intereosseus muscle and abductor digiti mimimus muscle MRC scores.

**Table 2:** Patient Characteristics.

Patient	Age	Gender	Disease duration from first symptom (months)	Disease duration from diagnosis and Riluzole start (months)	ALSFRSr	Average hand MRC (0-5)^1^	El Escorial stage
1^2^	67	Male	25	12	33	1.6	Definite ALS
2^2^	54	Male	31	22	23	0	Probable ALS
3^2^	68	Male	26	3^3^	43	5	Possible ALS
4	67	Male	57	13	41	4.6	Probable ALS
5^2^	75	Male	31	<1^4^	39	3.2	Probable ALS
6^5^	73	Male	25	2	43	4	Probable ALS
7	66	Female	9	5	46	5	Possible ALS
8	61	Male	19	12	41	4.6	Possible ALS
9^5^	48	Male	13	8	45	4.2	Possible ALS
10	70	Female	14	6^4^	34	3.2	Probable ALS
11	70	Male	15	3	39	4.2	Probable ALS

^1^Average MRC (range 0-5, with 5 being normal muscle power) of finger extension, finger flexion, thumb abduction, first dorsal intereosseus muscle and abductor digiti mimimus muscle on the contralateral side to the motor voxel placement. ^2^Treated with SSRIs. ^3^Taking Riluzole in reduced dosage 50 once daily. ^4^Not taking Riluzole. ^5^Unable to complete MRS.

The plan was initially to recruit a group of healthy age-matched subjects. Unfortunately, this was not possible due to a scanner breakdown. Instead, a group of 10, age comparable, healthy subjects, (7 male, mean age 65(range 58–73)) who had been scanned as part of a prior study^23^, conducted at the same time (April 2016), were included as controls. Healthy subjects were excluded if they had a history of major neurological or psychiatric disease, and if they were taking medication known to affect cortical neurotransmitter tone.

### Data acquisition

**Figure 2:**
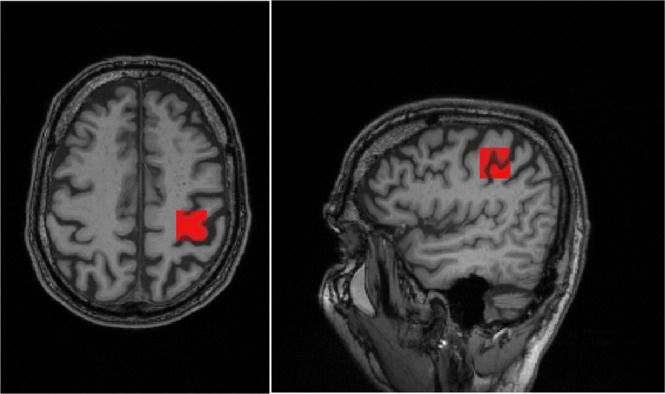
Example of voxel placement in the hand area of the primary motor cortex overlaid on MPRAGE T1.

All participants were scanned on a 3 T Siemens Trio (Erlangen, Germany) using a 32-channel head coil. A T1-weighted anatomical MPRAGE scan (TR/TE = 2420/4.6 ms, 1 mm isotropic resolution), was first acquired, and used to guide placement of a 2.5 × 2 × 2 cm MRS voxel in the occipital lobe, and a 2 × 2 × 2 cm MRS voxel in the hand area^24^ of the primary motor cortex (see Fig. 2).

MRS data were acquired using the ultrashort-TE Spin Echo Full Intensity Acquired Localized (SPECIAL) sequence^12^ (TR/TE = 4000/8.5 ms) with 128 and 154 averages acquired for the occipital and motor regions, respectively. For patients with ALS the voxel was placed in the contralateral hemisphere to the side of symptom onset in the arm or leg. For patients with bulbar onset or bilateral symptoms at onset, the voxel was placed in the left hemisphere. Prior to MRS, shimming was performed using FASTESTMAP (Fast Automatic Shim Technique using Echo-planar Signal readouT Mapping Along Projections)^25^. An unsuppressed water reference scan was acquired from each region for water scaling and eddy-current correction. Two patients were unable to complete the MRS scans due to respiratory problems and neck pain, respectively.

Due to failure of the gradient system the scanner was upgraded and out of service for several months. Consequently, prospectively selecting a group of age-matched healthy control subjects was not possible. As an alternative, a group of age comparable healthy subjects, recently scanned as part of a different study, were included for comparison. The group was scanned using the same scanner during the ALS experiment (April 2016), using the same MRS sequence, identical motor cortex voxel placement, albeit slightly fewer averages (146). For healthy subjects a number of left/right hemisphere voxels identical to the patient group was chosen for comparison. Unfortunately, we had no prior MRS data from an occipital voxel in a comparable age group.

### Data analysis

#### MR analysis

Motor voxel MRS data from ALS patients and healthy subjects were anonymized and blinded before analyses.

Pre-processing of the raw spectroscopy data was done in MATLAB (2017b, The MathWorks Inc., Natick, MA, 2017) using FID-A^26^. First, motion-corrupted averages were removed, followed by frequency and phase drift correction using spectral registration^27^. Spectral averages were then combined, followed by zero- and first order phase correction. Subsequently, the processed spectra were analysed in LCModel (v6.3, Provencher, 1993) to fit and estimate metabolite concentrations. The range of LCModel was set to a chemical shift of 0.2 to 4.2 ppm. The water unsuppressed scan was also utilized by LCModel for water scaling and eddy current correction. The data was fitted using a basis set that was simulated using the FID-A simulation toolkit^26^, and all pulses were simulated as instantaneous rotations. The final metabolite concentrations and metabolite/Creatinine ratios were then used for further statistical analysis.

Data quality was checked visually as well as by LCModel output of SNR, linewidth and fit uncertainty of metabolites values as measured using the Cramer-Rao Lower Bound of uncertainty (CRLB).

To correct for differences in grey matter content within the voxel, which is an issue of particular concern in neurodegenerative conditions such as ALS, the T1 scan was segmented using SPM12 (http://www.fil.ion.ucl.ac.uk/spm/), and the fractions of grey matter (GM), white matter (WM) and cerebrospinal fluid (CSF) within the MRS voxel were calculated.

NAA + N-acetylaspartylglutamate(NAAG) was expressed as tNAA, glycerophosphocholine(GPC) + phosphocholine(PCho) was expressed as tCho and creatine + phosphocreatine was expressed as tCr.

#### Statistics

Motor voxel MRS data were compared between ALS patients and healthy subjects using two-tailed Students t-tests. Associations between clinical measures and MRS findings were tested using standard Pearson product-moment correlation (r). For metabolites that differed significantly between patients and healthy subjects, ratios corrected for GM content in the voxel were calculated by dividing the values by the faction of GM in the voxel. For metabolites that correlated significantly with clinical outcome measures, correlation analysis were repeated with GM as an extra explanatory variable to test whether the correlation was explained by changes in GM content. A p-value below 0.05 was considered significant.
